# Laparoscopic treatment of congenital portosystemic shunts with portal pressure measurement and portal angiography in 36 dogs

**DOI:** 10.3389/fvets.2024.1291006

**Published:** 2024-02-28

**Authors:** Jin Shigemoto, Yasuyuki Kaneko, Mitsunobu Kawazu, Kiyokazu Naganobu, Shidow Torisu

**Affiliations:** ^1^Oji Pet Clinic, Tokyo, Japan; ^2^Laboratory of Companion Animal Surgery, Department of Companion Animal Clinical Sciences, School of Veterinary Medicine, Rakuno Gakuen University, Hokkaido, Japan; ^3^Miyazaki University Veterinary Medical Teaching Hospital Laboratory, Miyazaki, Japan; ^4^Animal Minimally Invasive Surgery Team (Amist), Oji Pet Clinic (Animal Minimally Invasive Center), Tokyo, Japan

**Keywords:** canine, congenital, laparoscopic surgery, portal pressure, portal vein angiography, portosystemic shunt

## Abstract

**Introduction:**

Laparoscopic surgery is used for canine congenital extrahepatic portosystemic shunts (CEHPSS). However, outcomes of laparoscopic surgery involving simultaneous portal vein angiography and portal pressure measurement to attenuate or completely occlude the shunt vessel in canines remain unclear. This study aimed to evaluate outcomes and complications of laparoscopic portosystemic shunt occlusion (LAPSSO) for CEHPSS.

**Methods:**

Between June 2014 and March 2021, data on dogs undergoing cellophane banding (CB) and complete occlusion of laparoscopically treated congenital extrahepatic port shunts were collected from hospital records. Cases in which complete occlusion was laparoscopically performed, or a CB was used for gradual occlusion were included. A total of 36 dogs (14 males; median age 32.5 months [range, 5–99] with median body weight, 4.2 kg [range, 1.5–7.9]) that underwent LAPSSO for CEHPSS were included. All the dogs underwent computed tomographic angiography (CTA), and data on blood and radiological examinations were collected. Shunt vessel morphology was categorized using CTA findings. Portal pressure measurements and portal angiography were performed by accessing mesenteric and splenic veins in 30 and 6 cases, respectively.

**Results:**

The most common shunt types were spleno-phrenic shunts 16/36 (44.4%), followed by spleno-azygos 9/36 (25.0%), spleno-caval 4/36 (11.1%), right gastric-caval 6/36 (16.6%), and right gastric-caval with caudal loop shunts 1/36 (2.7%). The median portal pressure after complete occlusion was 11.5 mmHg (range, 4–16); portal pressures in the two dogs undergoing CB attenuation were 22 and 24 mmHg. The median operating time in the dogs with right (*n* = 25) and left (*n* = 11) recumbent positioning was 55 min (range, 28–120) and 54 min (range, 28–88), respectively. One dog had pneumothorax due to injury to the diaphragm. Another dog developed postoperative hypernatremia and succumbed 5 h post-procedure. Nevertheless, no other dogs exhibited signs of portal hypertension within 72 h. Blood tests and abdominal ultrasounds performed 1–2 months postoperatively revealed no residual shunts.

**Discussion:**

LAPSSO, coupled with portal pressure measurement and portal angiography, was shown as safe and effective approach that facilitated successful occlusion of CEHPSS. Further large-scale prospective studies and analyses of perioperative complications are needed.

## Introduction

Canine congenital portosystemic shunts involve the shunting of portal blood into the caudal vena cava or azygos vein, leading to the systemic circulation of substances such as plasma ammonia. This can result in conditions such as hepatic encephalopathy, urinary calculi, coagulation disorders, and delayed development (1–10). Congenital portosystemic shunts are classified as extrahepatic portosystemic shunts and intrahepatic portosystemic shunts (3, 8, 9). The prevalence of congenital extrahepatic portosystemic shunts (CEHPSS) is 0.06–0.2% and is most common in small purebred dogs such as Yorkshire Terriers, Malteses, Miniature Schnauzers, and Cairn Terriers (7). CEHPSS can adversely affect the quality of life of the animals, and treatment modalities with low risks and complications are limited, if relevant.

Occlusion of the shunt vessel to restore physiologic blood flow to the liver has shown promise in improving the quality of life or even curing dogs with CEHPSS. Laparotomy is the standard surgical procedure for CEHPSS in dogs, with the goal of completely occluding the shunt vessels in the inflow area leading to the systemic circulation (1–5). The main anatomical types of CEHPSS include spleno-caval, spleno-azygos, right gastric-caval and right gastric-azygos shunts (6–10). Shunt vessel identification is achieved by making a large midline incision from the xiphoid process to the anterior margin of the pubic bone and placing traction or pressure on the gastrointestinal tract (8–10). The confirmed shunt vessel can be completely occluded using a surgical thread or partially occluded by placing a cellophane band (CB) or an ameroid ring constrictor (ARC) (11–15). When performing complete occlusion, portal pressure is measured in the mesenteric vein to prevent portal hypertension due to rapid changes in portal pressure. Accordingly, the decision to occlude the shunt vessel completely or partially is determined based on the portal pressure measurement, changes in the color of the pancreas and gastrointestinal tract, and the degree of peristaltic motion (1, 5). Moreover, intraoperative portal angiography can be performed to confirm the shunt vessel. Recent field advances such as the dissemination of preoperative CT and shunt vessel classifications have eliminated the need for performing portal angiography to confirm the shunt vessel (1, 11–15). Generally, when using CB or ARC, post-surgery portal pressure measurement and intraoperative portal venography are forgone, as occlusion occurs gradually (5, 6, 11–15). This approach, however, has led to complications such as shunt vessel misidentification, postoperative recanalization, and residual, shunt vessels (11–15).

Various laparoscopic surgery approaches, which are less invasive, less painful, and less inflammatory than open procedures, have been recently reported for dogs (16–18). Nonetheless, there are no reports to date of laparoscopic surgery combining simultaneous portal vein angiography and portal pressure measurement for shunt vessel identification and partial or complete occlusion (19–24). The purpose of this study was to describe the technique, outcomes and complications of LAPSSO combined with portal pressure measurement and portal angiography as well as to evaluate the outcomes and complications.

## Materials and methods

### Inclusion criteria

Medical records of dogs undergoing LAPSSO for CEHPSS at the Oji Pet Clinic from June 2014 to March 2021 were reviewed. Inclusion criteria were cases with laparoscopic portal venous pressure measurement, portal angiography, complete occlusion with surgical thread, and/or attenuated with CB. All dogs were classified for the shunt type by computed tomography angiography (CTA). The follow-up period was from 1 to 2 months. From the medical records, age, weight, clinical signs, anatomical shunt type, method of occlusion, measurement of portal venous pressure, and portal angiography. Operative time, complications, and clinical outcomes were included. All owners whom's underwent LAPPSO provided written consent.

### Diagnostic evaluation

Data regarding blood ammonia and total serum bile acids pre- and post-prandial, as well as blood counts, blood chemistry, abdominal X-ray, and abdominal ultrasound (AUS), were collected either at the referring clinic or our clinic. Based on these results, CTA was performed on all dogs with suspected CEHPSS to characterize the shunt vessel morphology. The approach of LAPSSO with portal pressure measurement and portal angiography was planned using images from CTA (5–10).

### Anesthesia

Midazolam (0.2 mg/kg IV or subcutaneously; Dormicum; Astellas Pharma Inc., Tokyo, Japan) and atropine (0.01 mg/kg IV or subcutaneously; atropine sulfate injection 0.5 mg; Nipro ES Pharma Co., Ltd., Osaka, Japan) were used for premedication. Anesthesia was induced using propofol (6–10 mg/kg IV; propofol 1%; Intervet K.K., Tokyo, Japan), maintained with 100% oxygen and isoflurane (Isoflu; DS Pharma Animal Health Co., Ltd., Osaka, Japan) or sevoflurane (Sevofrane; Pfizer Japan Inc., Tokyo, Japan), and managed using an anesthesia monitor.

Epidural morphine (0.01 mg/kg; morphine hydrochloride injection 10 mg; Daiichi Sankyo Co., Ltd., Tokyo, Japan) or intravenous remifentanil (0.3–0.7 μg/kg/min IV; Ultiva 2 mg; Janssen Pharmaceutical K.K., Tokyo, Japan) was administered as an analgesic at a continuous infusion rate. Blood pressure was directly measured by placing an implant in the dorsal pedis artery after anesthesia induction. Dopamine (5 μg/kg/min IV; dopamine HCl 100 mg; Teva Takeda, Ltd., Nagoya, Japan) was administered at a continuous infusion rate, as needed, to maintain a mean arterial pressure ≥ 60 mmHg. If spontaneous breathing did not stop, rocuronium (0.5 mg/kg IV; Eslax 25 mg; MSD K.K., Tokyo, Japan) was administered.

### Surgical procedure

A 5 mm 30° laparoscopic telescope (Hopkins II Telescope; Karl Stortz, Tuttlingen, Germany) was attached to a video camera (Telecam SLII; Karl Stortz) and light source (Xenon Nova 300; Karl Stortz), with manipulations being recorded while adjusting the light intensity. The body position was decided according to the approach site determined based on the CTA results.

**Figure 1:**
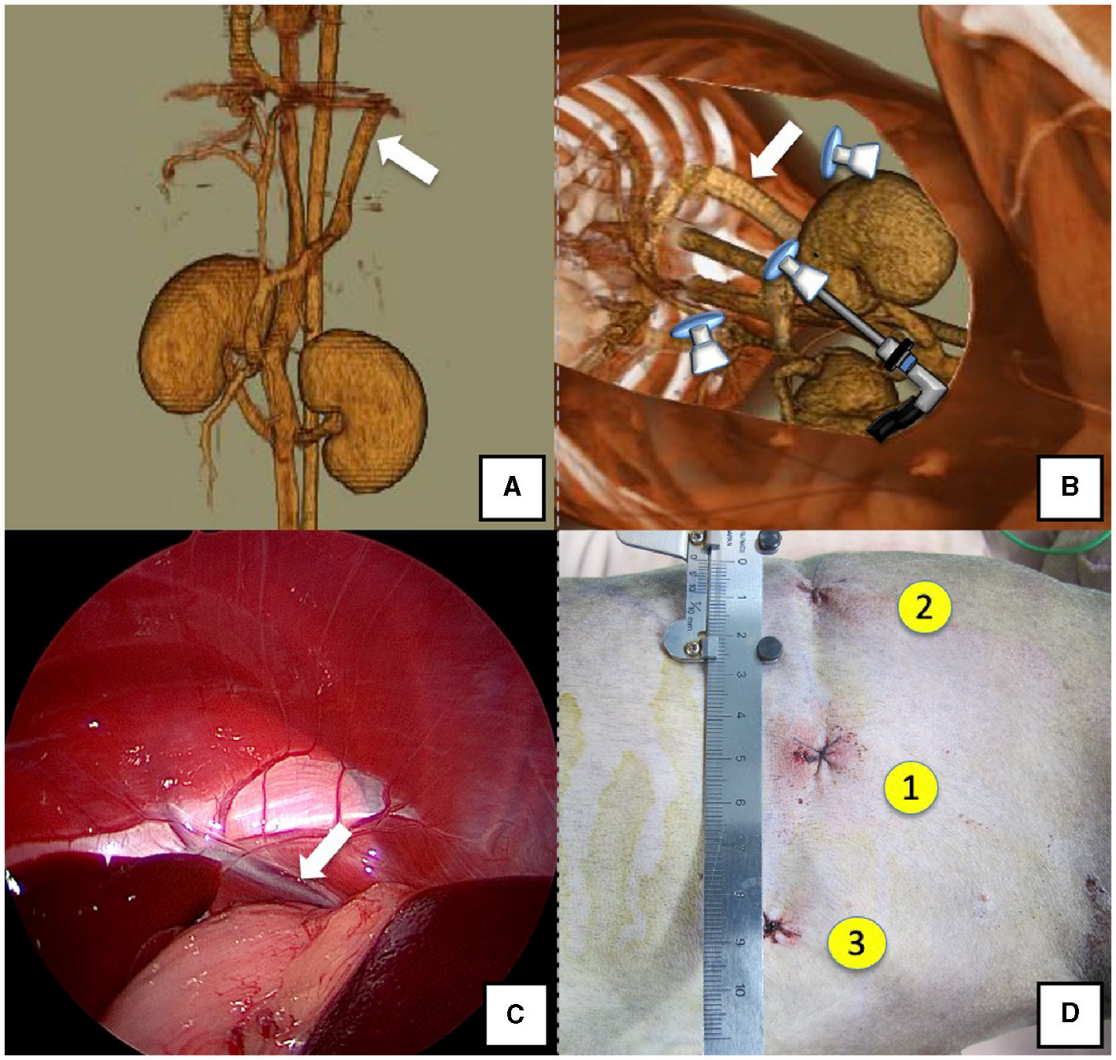
Spleno-phrenic shunt (white arrow). **(A)** 3D-CT angiography image. **(B)** 3D-CT image of the port locations from reconstructed right recumbent CT images. **(C)** Laparoscopic image of the shunt vessel and approach. **(D)** Actual surgical wounds and port placement order. There are surgical wounds for only three ports because the splenic vein was used percutaneously. 3D-CT, three-dimensional-computed tomography.

**Figure 2:**
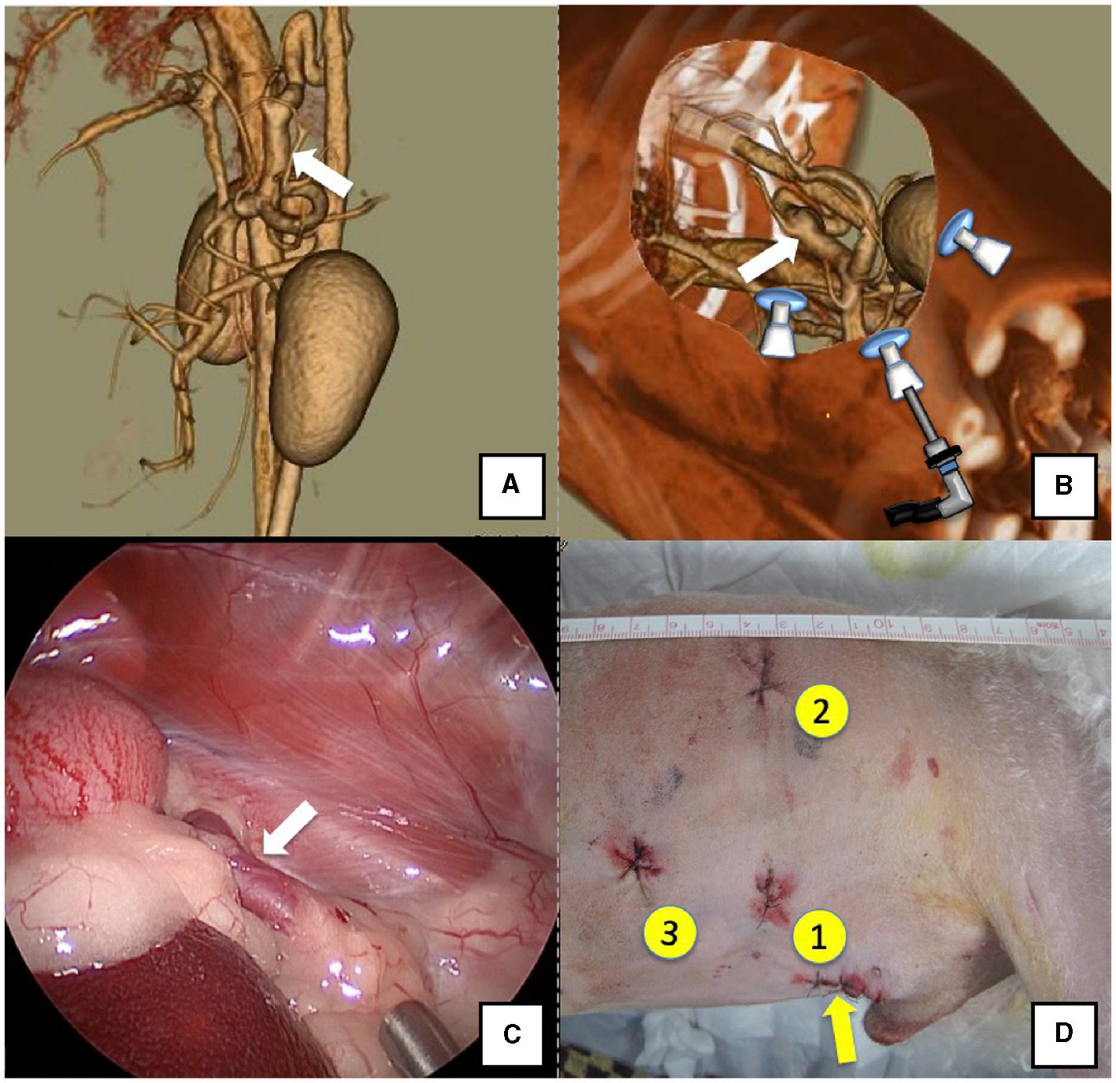
Spleno-azygos shunt (white arrow). **(A)** 3D-CT angiography image. **(B)** 3D-CT image of the port locations from reconstructed right recumbent CT images. **(C)** Laparoscopic image of the shunt vessel and approach. **(D)** Actual surgical wounds and port placement order. Yellow arrow: location where the LAP PROTECTOR was placed. 3D-CT, three-dimensional-computed tomography.

**Figure 3:**
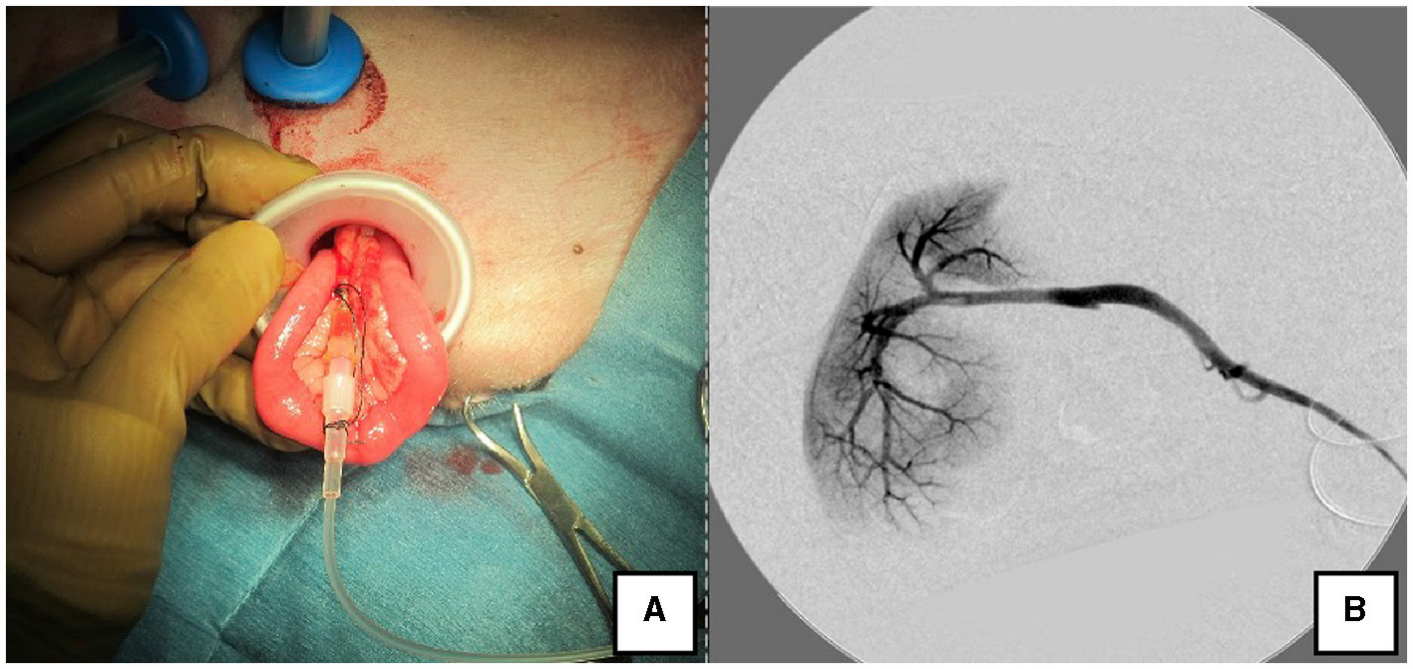
Portal angiography and portal pressure measurement using LP. **(A)** The jejunum was pulled outside the body using the LP, and an indwelling needle was inserted to measure portal pressure. **(B)** Portal vein angiography during shunt ligation with a C-arm. LP, LAP PROTECTOR.

**Figure 4:**
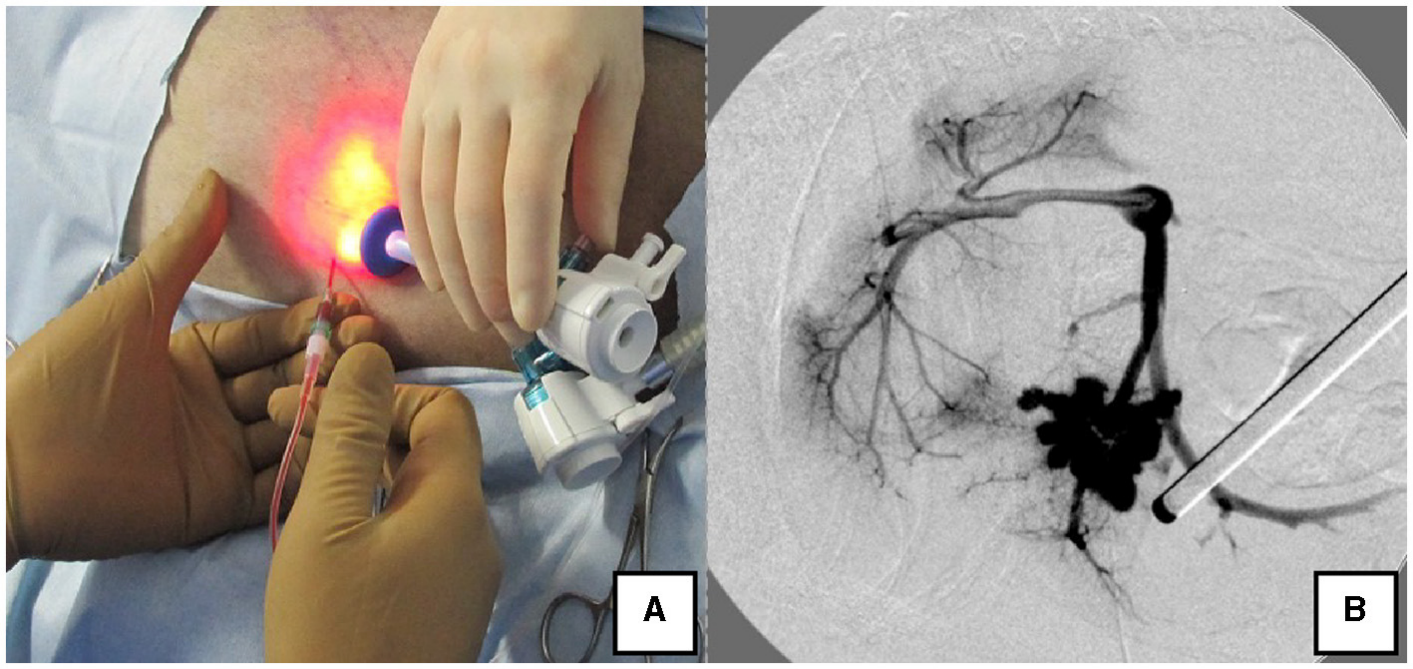
Portal pressure measurement and portal angiography via the splenic vein. **(A)** Portal pressure was measured by percutaneously inserting an indwelling needle into the spleen while confirming the spleen with the laparoscope. **(B)** Portal angiography during shunt ligation with a C-arm.

The right lateral position was used for cases lacking shunt vessels in the epiploic foramen, including splenic-pheric (Figures 1A, B) and splenic-azygos (Figures 2A, B) shunts. Cushions and towels were placed against the back. The shunt position was simulated in advance using CTA, and the site of port No. 1 cannula (Kii Sleeve with Advanced Fixation, Applied Medical, Rancho Santa Margarita) was marked using a medical oil-based marker. Subsequently, a 5-mm-diameter port No. 1 was inserted in the cranial and midline directions of the renal head side of the paralumbar fossa using the Hasson open technique, followed by the establishment of pneumoperitoneum using CO_2_ at 8-10 mmHg pneumoperitoneal pressure. Next, the camera was inserted into the abdominal cavity via port No. 1 and used to determine the position of port No. 2, which was approximately 3 cm to the right and slightly cranial to port No. 1 and was used to insert forceps (Tear Drop Forceps; Olympus, Hamburg, Germany) for peeling. Port No. 3, used to insert forceps (flat jaw insert for thoracic surgery; Olympus, Tokyo, Japan) for traction on the organs surrounding the shunt, was created to the left and cranially to port No. 1, with a distance of ≥3 cm. Ultimately, three ports were placed in the paralumbar fossa (Figures 1D, 2D). The shunt vessel was easily confirmed by compressing the spleen and stomach to the left using a palpation probe retractor (Palpation Probe; Karl Storz; Figures 1C, 2C). The shunt was carefully detached and then secured with a 10 cm piece of 3-0 braided nylon thread. The mesenteric or splenic vein was used for portal pressure measurements and portal angiography. For the mesenteric vein, a small incision (2–3 cm) was made, followed by the attachment of a silicone single-hole endoscopic retractor (LAP PROTECTOR [LP]; Hakko, Nagano, Japan). Part of the gastrointestinal tract was removed from the body to measure the portal pressure and perform portal angiography on the mesenteric vein (Figure 3). When performing trans-splenic portal angiography and portal pressure measurements, the skin was subcutaneously pierced, and the spleen was pierced within the body using an indwelling needle, followed by the measurement of the portal pressure at the point of blood reflux while confirming bleeding using an endoscope (Figure 4) (22–24). Next, temporary occlusion was performed by placing traction on the braided nylon thread used to secure blood flow in the shunt vessel, followed by portal pressure measurement and portal angiography (Figures 3, 4). The shunt vessel was temporarily blocked, followed by CB attenuated or complete occlusion after confirming that there was no shunt vessel misidentification on portal angiography as well as portal pressure measurement and determination of the color tone of the pancreas and intestinal tract. If necessary, concurrent with cystotomy for bladder calculus, neutering was performed. Finally, a liver biopsy was performed. The animal was postoperatively checked for abdominal bleeding or deterioration in the color of the gastrointestinal tract, followed by removal of the ports and routine wound closure (Figures 1D, 2D).

**Figure 5:**
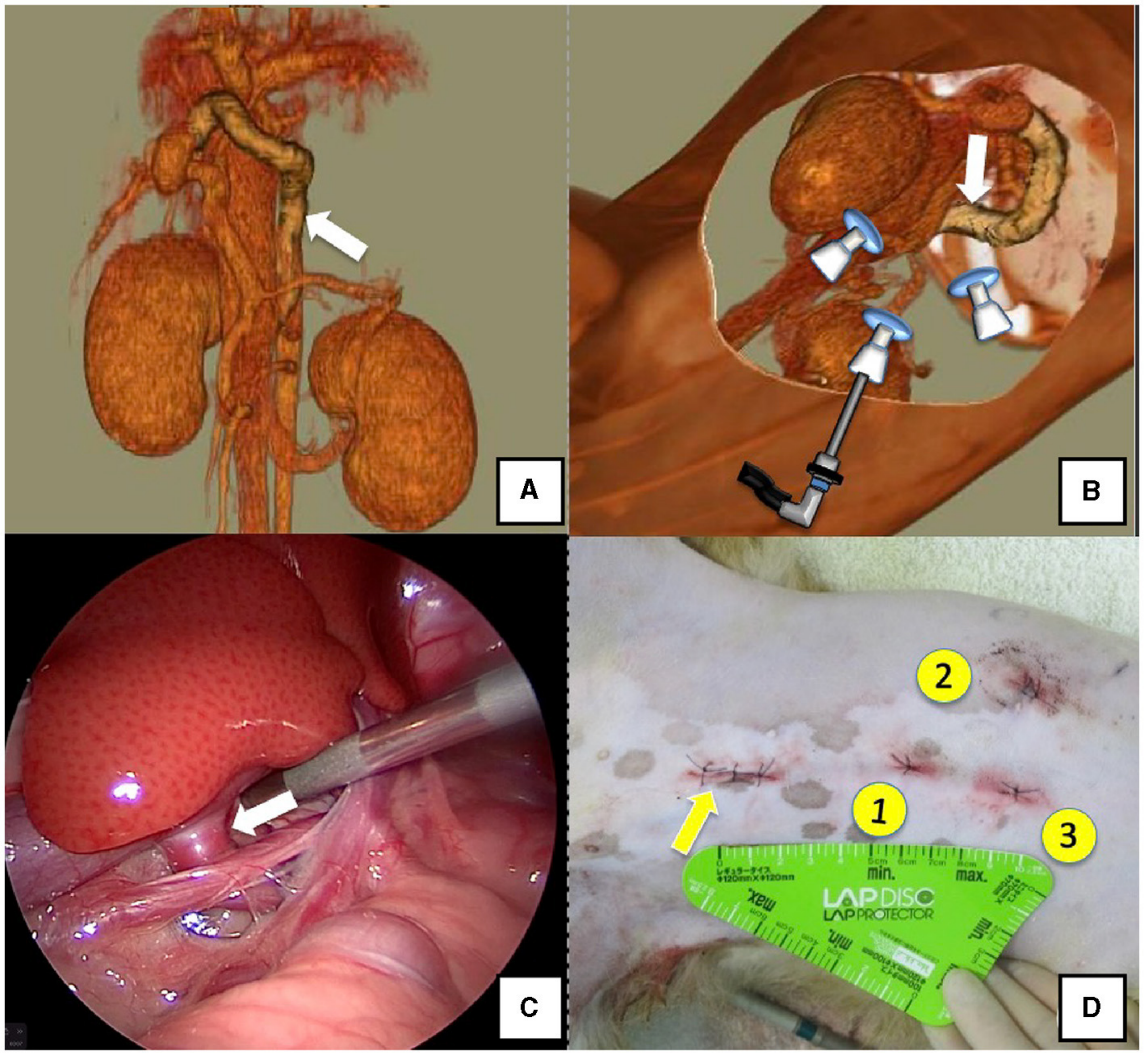
Right gastric-caval shunt (white arrow). **(A)** 3D-CT angiography image. **(B)** 3D-CT image of the port locations from reconstructed left recumbent CT images. **(C)** Shunt vessel and approach as seen from the laparoscope. **(D)** Actual surgical wounds and port placement order. Yellow arrow: location where the LAP PROTECTOR was placed. 3D-CT, three-dimensional-computed tomography.

Dogs with shunt vessels located in the epiploic foramen, including right gastric-caval, spleno-caval, and right gastric with caudal loop shunts, were placed in the left lateral recumbent position (Figures 5A, B). These shunts were occluded at the epiploic foramen at the inflow into the caudal vena cava; accordingly, the duodenum and pancreas were moved to the left to achieve this. We placed a cushion on the back side during immobilization or tilted the operating table by ≈10° toward the surgeon to displace the gastrointestinal tract to the ventral side, which allowed surgical field visualization. Port No. 1 was established using the Hasson open technique to allow observation of the right adrenal gland from the right paralumbar fossa. The camera was inserted into port No. 1 for observation of the shunt vessel after establishment of pneumoperitoneum using 8–10 mmHg pneumoperitoneal pressure. Next, port No. 2 was created ≥3 cm to the left of port No. 1 to observe the abdominal cavity. A palpation probe was inserted to the left of the caudal vena cava from port No. 2; moreover, shunt blood flow into the caudal vena cava was confirmed by slightly lifting the caudal vena cava to the right (Figure 5C). Port No. 3 was created ≥3 cm to the right of port No. 1 to allow detachment and ligation of the shunt vessel. The shunt vessel was detached after installation of these three ports and secured with a 10 cm 3-0 braided nylon thread. Given the difficulty in observing the spleen in the left recumbent position, portal pressure measurement and portal venography were performed using a retractor LP. After securing the shunt vessel, a 2.0–3.0 cm paramedian incision was made caudally to the umbilicus, a retractor LP was inserted, and the jejunum was pulled outside the abdominal cavity. As aforementioned, an indwelling needle was placed in the mesenteric vein for portal pressure measurement, with temporary halting of pneumoperitoneum, as it affects these measurements. Next, the previously secured shunt vessel was temporarily occluded, followed by repeated portal pressure measurements and portal angiography using the C-arm. Subsequently, the indwelling needle was removed from the mesenteric vein, and the intestinal tract was returned to the abdominal cavity. A special cover (E-Z Access; Hakko) was placed on the retractor LP. Next, pneumoperitoneum was established again, followed by complete occlusion or CB attenuated of the secured shunt vessel using suturing forceps (Needle Holder; Olympus). If necessary, concurrent with cystotomy for bladder calculus, neutering was performed. Finally, intra-abdominal bleeding and gastrointestinal tract color were checked. The camera was removed while checking for intra-abdominal bleeding and organ damage, followed by wound suturing (Figure 5D).

### Postoperative care

After surgery, the animals were placed in intensive care for 72 h to monitor for postoperative complications such as hypoglycemia, post-attenuation neurological signs (PANS), and portal hypertension (25–27). Blood glucose levels were measured every hour; AUS was performed at 8–24-h intervals; and light, eyelid, and menace reflexes were checked. Oral feeding was started at ≈ 2 h postoperatively. If hypoglycemia was observed (glucose ≤ 50 mg/dL), 50% glucose was administered orally, occasionally with prednisolone 0.5 mg/kg and dexamethasone 0.1 mg/kg. Levetiracetam (20 mg/kg, every 8 h) was administered for 7 days postoperatively.

### Ethical consideration

Ethical approval was not required because the animals were handled in accordance with local laws and institutional requirements for research involving animals. Written informed consent for the participation of animals in this study was obtained from their owners. Our study is a retrospective study. In the present study, we performed laparoscopic portosystemic shunt vascular occlusion in dogs. Previously reported laparoscopic portosystemic shunt occlusion procedures did not include portal vein pressure measurement and portal vein angiography, resulting in misidentification of the shunt vessel. When we performed laparoscopic portosystemic shunt surgery, we opened the abdomen through a small incision and placed a lap disk as in open surgery to measure portal vein pressure and perform portal angiography. The laparoscopic technique, in which a small incision is made in the abdominal wall, a lap disk is placed, and the digestive tract is removed from the body cavity to perform a full-layer biopsy of the digestive tract, is also performed as a normal biopsy procedure, and the safe removal of the digestive tract from the body cavity under laparoscopy is not a procedure requiring ethical review. The percutaneous perforation of the spleen for portal venography is also a procedure that has already been reported and is not a special procedure. The novelty of this study is that it demonstrated that portal venous pressure measurement and portal angiography can be performed safely by combining conventional techniques in laparoscopic portosystemic shunt vascular occlusion. Therefore, we determined that ethical review was not necessary because the study was simply a combination of conventional techniques to perform a safer procedure.

## Results

### Cases and clinical findings

Laparoscopic surgery was performed in 36 dogs of CEHPSS during the study period. Thirty-four dogs underwent complete occlusion, and 2 dogs underwent surgery using a CB. There were 14 males (9 castrated) and 22 females (12 sterilized). The median age and weight were 32.5 (range 5–99) months and 4.2 (range 1.5–7.9) kg, respectively. The breeds included seven Miniature Schnauzers, seven Toy Poodles, six Yorkshire Terriers, three Malteses, three Shih Tzus, two Shiba Inus, and one each of mixed breed, Italian Greyhound, Welsh Corgi, Bichon Frise, Chihuahua, Papillon, Pomeranian, and Miniature Dachshund.

The main preoperative clinical findings included bladder calculi accompanied by hematuria and frequent urination (12 dogs), neurological signs indicative of hepatic encephalopathy such as vomiting and drooling (12 dogs), and seizure with loss of consciousness (4 dogs). Liver-specific nutrition was prescribed in case of preoperative hyperammonemia. All dogs received lactulose (0.5–1 mL/kg, every 12 h) and a blanched chained amino acid supplement (0.6 g/kg; Vercure Liv., NST, Saitama, Japan).

### Diagnostic and surgical findings

Table 1 provides details on the anatomical shunt types, operation time for occlusion, portal pressure at occlusion, and complications. The shunt types diagnosed by CT in this study were as follows: spleno-phrenic shunts 16/36 (44.4%), followed by spleno-azygos 9/36 (25.0%), spleno-caval 4/36 (11.1%), right gastric-caval 6/36 (16.6%), and right gastric-caval with caudal loop shunts 1/36 (2.7%). In all cases, the portal vein pressure was measured laparoscopically, and the shunt vessel was confirmed by contrast and ligated or banded. The operating time was defined as the time from incision to complete occlusion or CB attenuation after portal pressure measurement and portal angiography. The median operating time in the dogs with right (*n* = 25) and left (*n* = 11) recumbent positioning was 55 min (range 28–120) and 54 min (range 28–88), respectively. Portal vein pressure measurements and LP portal angiography were performed in the mesenteric and splenic veins of 30 and 6 dogs, respectively. Dogs with a median portal pressure of 11.5 mmHg (range 4–16) after temporary occlusion were classified as completely occluded; two dogs with CB attenuation exhibited pressures of 22 and 24 mmHg (Table 1).

**Table 1:** Anatomical shunt types and positions, portal pressure measurement and portal angiography methods, preoperative and postoperative patient characteristics, and time to occlusion in laparoscopic portosystemic shunt occlusion.

**Shunt type**	**Patient recumbency**	**Portal vein pressure after temporary blockade**	**Method of portal angiography**	**CB or complete occlusion**	**Complication**
Spleno-pherenic (*n* = 16)	Right lateral	11 (4–16) mmHg	LP (*n* = 13) SP (*n* = 3)	Complete (*n* = 16)	PANS (*n* = 1)
Spleno-azygos (*n* = 9)	Right lateral	12 (9–24) mmHg	LP (*n* = 6) SP (*n* = 3)	Complete (*n* = 8) CB (*n* = 1)	PANS (*n* = 1) Hypernatremia and died 5 h later (*n* = 1) Pneumothorax during detachment (*n* = 1)
Spleno-caval (*n* = 4)	Left lateral	11 (9–14) mmHg	LP (*n* = 4)	Complete (*n* = 4)	None
Right gastric -caval (*n* = 6)	Left lateral	12 (11–22) mmHg	LP (*n* = 6)	Complete (*n* = 5) CB (*n* = 1)	None
Right gastric-caval with caudal loops (*n* = 1)	Left lateral	7 mmHg	LP (*n* = 1)	Complete (*n* = 1)	None

LP, portal pressure measured, and portal angiography performed with LAP PROTECTOR; SP, portal pressure measured and portal angiography performed with percutaneous splenic puncture, PANS, post attenuation neurological signs.

### Complications and clinical outcomes

There was no conversion to open surgery due to an inability to control bleeding by laparoscopy or failure to identify the shunting vessel. Intraoperative complications included minor bleeding in 5 dogs (13%) due to dissection of shunt vessels, but bleeding could be managed. Another dog (3%) had pneumothorax due to an injury to the diaphragm. This was a iatrogenic pneumothorax developing while dissecting a shunt flowing into an odd vein. A capnometer abnormality was noted at this time. An increase in airway pressure was noted and a decrease in transcutaneous arterial blood oxygen saturation (SpO_2_) was observed. A single trocar was immediately added to the chest and carbon dioxide in the thoracic cavity was drained. The dog was maintained at 5 mmHg pneumoperitoneum and which allowed to dissect and manipulate the shunting vessel while relieving pressure in the thoracic cavity. This dog had a portal pressure of 24 mmHg at the time of temporary occlusion, so a CB was placed. Therefore, it was necessary to spend a considerable amount of time, 120 min, before partial occlusion. Considering the fact that the occlusion site of a shunt vessel that flows into a diaphragmatic vein or odd vein is often in contact with the diaphragm, dogs with lung disease or adrenal problems should be carefully monitored. No complications such as portal hypertension, ascites, or residual shunts were observed at 72 h postoperatively. A chest tube was inserted into the thoracic cavity to reduce thoracic pressure, which allowed the shunting vessel to be dissected at the end of the procedure. The dog was fitted with a CB. In all dogs, LAPPSO could be performed with portal pressure and portal angiography. Eight dogs (20%) had subcutaneous bleeding postoperatively. One dog (2.7%) with complete occlusion developed postoperative hypernatremia and died after 5 h. This dog had no perioperative portal hypertension or ascites. Other complications included biliary obstructive disease in one case at 72 h postoperatively and elevated bilirubin levels, which improved on the eighth postoperative day and the patient was discharged. All other dogs underwent clinical examination and AUS at 6-h postoperative intervals until 72 h. With the exception of the dog that died, none of the 35 dogs exhibited severe abdominal pain or ascites retention due to portal hypertension within 72 h. This was attributed to the implementation of portal venous pressure measurements and portal angiography.

Blood glucose levels were measured every 2 h postoperatively. Four dogs (10%) had hypoglycemia below 50 mg/dl within 24 h postoperatively. These dogs were treated with oral or intravenous glucose and recovered. Two dogs developed PANS after the occlusion. One of them had a mild seizure after discharge and was treated at a local hospital for epilepsy. The other had a severe seizure 77 h later, lost vision immediately, and developed aspiration pneumonia on the fifth day, but was discharged without improvement in blindness after 23 days of treatment; one dog had preoperative neurological signs that persisted postoperatively, and this dog did not develop PANS during a 2 months followed-up period. However, one dog with persistent neurologic signs and one with PANS required continued epilepsy medication. As postoperative indicators, total bile acid levels and ammonia levels were measured in 30 dogs within 1 to 2 months after the surgery. In 26 (86.6%) dogs, blood anmonia levels returned to normal within 1 to 2 months after the surgery (pre-prandial median 31 μmol/mL, range 14–148 μmol/mL; post-prandial median 26 μmol/mL, range 9 - 128 μmol/mL). Serum bile acid levels had normalized 1–2 month after the surgery in 16 dogs (53.3%) with pre-prandial median 3.4 μmol/mL (range 0.1–65.9 μmol/mL) and post-prandial median 15.1 μmol/mL (range 0.9–87.7 μmol/mL). Abdominal ultrasound was performed within 1–2 months postoperatively in 34 dogs and showed no obvious residual ascites or shunts; one dog did not come in for suture removal or reexamination and could not be contacted; the general condition of 34 dogs was confirmed from medical records over 1 year after surgery. Thirty-five owners were interviewed by telephone between June 2022 and August 2022. Thirty four owners that could be contacted were interviewed. At the time of conducting the telephone interview, there were no fatal cases. All dogs whose owners were able to be interviewed said that their dog's clinical signs had improved and there were no signs of suspected recurrence.

## Discussion

This study demonstrates the feasibility of performing laparoscopic portal venous pressure measurement and portal angiography while occluding the shunt vessel in dogs with CEHPSS. There were no signs of portal hypertension, including ascites, intestinal edema, and abdominal pain in 35 of the dogs within 72 h postoperatively. The prognoses were relatively favorable. These findings indicate that LAPSSO procedure with portal pressure measurement and portal angiography is an option for treating CEHPSS.

LAPSSO requires preoperative CTA and 3D reconstruction of the CTA data to determine port sites and body positions, and also demands consideration of the occlusion method for each case. The average weight of dogs undergoing LAPSSO was 4.2 kg, and all cases involved small dogs. This is likely attributed to the high prevalence of CEHPSS in small dogs and the significant number of small dogs in Japan (3, 7).

The LAPSSO position was determined based on CTA findings. Specifically, based on the CTA results, the shunt occlusion site was the epiploic foramen in 11/36 (31%) dogs; accordingly, they underwent surgery in the left recumbent position. In the remaining 25/36 (69%) dogs, the shunt vessel blood flowed into the phrenic or azygos vein; therefore, the right recumbent position was used. Preoperative selection of the body position prevented intraoperative changes in the body position or a transition to open surgery. However, in cases with spleno-phrenic shunts, the shunt vessel begins from the left gastric vein and follows the dorsal stomach to connect with the phrenic vein, which runs through the diaphragm via the lesser stomach curvature. Accordingly, the occlusion site is very short and involves a risk of pneumothorax damaging the diaphragm (19, 21). In some cases, detachment of the shunt vessel took a relatively long time. Sometimes, it was difficult to identify shunt vessels connected with the azygos vein when the detachment site was behind the stomach and hidden by adipose tissue. In these cases, the shunt vessel could be observed and ligated by adjusting the inclination of the dog and moving the ports slightly to the caudal visualization of the azygos vein.

In previous studies in which CB or ARC were used in dogs, portal venous pressure measurement and portal angiography were not performed (11–15, 19–21). Instead, the shunt was temporarily occluded with CB, and portal hypertension was monitored by changes in pancreatic and intestinal color and increased peristalsis; CB and ARC are very useful occlusion modalities in dogs that cannot tolerate complete occlusion. However, there are many reports that CB and ARC result in residual shunt vessels postoperatively (11–15). Landon et al. reported incomplete occlusion in 3 (18.6%) out of 16 dogs operated for CB and multiple acquired shunts in 3 dogs (13). Nathan et al. reported complete occlusion in 13 (65%) out of 20 dogs operated for CB, while incomplete occlusion occurred in 7 dogs (14). The cause of shunt remnant in CB attenuated cases has not been clarified, but it has been suggested that misidentification of the shunt or problems with its location may play a role (13.14). Poggi et al. reported that intraoperative portal pressure measurement and portovenography are important, despite the challenges associated with these techniques, if possible, for complete occlusion (19–24). The operative time also tends to increase, thus increasing the burden. Laparoscopic portal vein shunting has also been reported to be difficult to measure portal venous pressure because of the difficulty in placing an invasive portal measurement device (20). In humans, portal pressure is measured after temporary occlusion of the shunt vessel; further, there have been reports of partial occlusion being performed owing to portal hypertension (28, 29). Mori et al. described measurement of retrograde portal pressure from the femoral vein via the shunt; however, dogs are too small for this procedure (3, 7). Therefore, we used a retractor LP for portal pressure measurement similar to the measurement procedure in open surgery. An LP is a stretchable small-incision silicone device for laparoscopic use that is available in various sizes. During the procedure, a small skin incision of about 2.0–3.0 cm was made, the jejunum was taken outside the abdominal cavity, and the LP was placed in the mesenteric vein. By attaching a special lid (EZ access) to the LP, it was possible to re-establish pneumoperitoneum and wrap a surgical thread or CB around the shunt to close it. In some dogs, bladder calculi were removed, or contraceptive surgery was simultaneously performed with LAPSSO using the LP holes. Accordingly, the retractor LP was considered a useful tool in this procedure.

Alternatively, portal pressure measurement and angiography can be performed by percutaneously placing a long indwelling needle in the spleen to simultaneously measure portal pressure and perform angiography of the splenic vein (SP) (24). This SP method was feasible only in the right recumbent position with clear visualization of the spleen in the abdominal cavity, no bleeding tendencies, and sufficient splenic thickness. SP was used in 6 of the 25 (24%) dogs held in the right lateral recumbent position. However, LP was used in the remaining cases where it was thought that the port would make contact with the indwelling needle placed in the spleen or owing to concerns regarding hemostasis. Therefore, while body position is a vital consideration when choosing the method for portal pressure measurement and contrast, it is equally crucial to take into account the patient's body size and propensity for bleeding. We suggest that intraoperative portal venous pressure measurement and identification of shunt vessels may reduce postoperative portal hypertension, ascites, and residual shunt vessels. Furthermore, even in dogs in which preoperative CTA findings predicted that complete occlusion was possible, some dogs had high portal pressures at the time of temporary occlusion of the shunt vessel. In such cases, if the portal vein pressure was greater than 16 mmHg, the method of occlusion was changed to partial occlusion with CB or surgical thread. Many factors influence intraoperative portal pressure, including systemic blood pressure and the location of the intestinal tract (1,4,5.). Therefore, mean arterial pressure was maintained above 60 mmHg, after which portal pressure was measured simultaneously. The portal pressure was adjusted to remain below 16 mmHg extensively.

In our study, both subjective and objective follow-up assessment methods were applied. Regarding objective assessment methods, 34 dogs underwent preoperative and postoperative ammonia and bile acid tests as well as abdominal ultrasonography. Two dogs with partial occlusion underwent blood tests after achieving complete occlusion. There was a postoperative improvement in ammonia and bile acid levels (30–32). Long-term follow-ups included in-person and telephone interviews with the owners regarding clinical findings and signs of recurrence; however, there were no cases of recurrence of clinical signs or complications.

There are several limitations to this study. First, the study had a small sample size. Because of the phenomenon of port interference and the possibility of forceps interference due to the narrow working space in the abdominal cavity. And there was only one institution and no multi-center study, which may have bias in case selection. Second, although follow-up was conducted for 2 months after treatment, CTA was not performed, and the accuracy of the study is problematic because the residual shunt vessels were confirmed by AUS at the time of reexamination. Detection of small shunt vessels is difficult, and the outcome may be somewhat less satisfactory. If possible, CTA can be performed at the postoperative checkup for more accurate evaluation. Third, the small number of studies of partial occlusions does not take into account complications and prognosis, and more studies are needed. A large prospective study is required to validate our findings, including complete occlusion, operative time, complications, and the conversion rate to laparotomy.

In conclusion, the results of the present study suggest that portal hypertension measurement and portal angiography can be performed in LAPSSO in CEHPSS and may reduce postoperative portal hypertension and residual shunt vessels and multiple shunts. Furthermore, the majority of reexamined dogs displayed the absence of residual shunts, recanalization, or signs of recurrence more than 1 year postoperatively. While LAPSSO with LP is more complex, invasive, and time consuming than conventional laparoscopic surgery, it holds the potential to enhance postoperative outcomes and prognosis.

## Data availability statement

The original contributions presented in the study are included in the article/supplementary material, further inquiries can be directed to the corresponding author.

## Ethics statement

Ethical approval was not required because the study was a retrospective study and the animals were handled in accordance with local laws and institutional requirements for research involving animals. Written informed consent was obtained from the owners for the participation of their animals in this study.

## Author contributions

JS: Conceptualization, Formal analysis, Investigation, Writing—original draft, Writing—review & editing. YK: Project administration, Writing—review & editing. MK: Writing—review & editing, Resources. KN: Project administration, Writing—review & editing. ST: Writing—review & editing, Project administration.
